# The application of the WSES classification system for open pelvic fractures—validation and supplement from a nationwide data bank

**DOI:** 10.1186/s13017-022-00434-y

**Published:** 2022-05-27

**Authors:** Pei-Hua Li, Ting-An Hsu, Yu-Chi Kuo, Chih-Yuan Fu, Francesco Bajani, Marissa Bokhari, Justin Mis, Stathis Poulakidas, Faran Bokhari

**Affiliations:** 1 Department of Trauma and Emergency Surgery, Chang Gung Memorial Hospital, Chang Gung University, Taipei, Taiwan; 2 Department of Trauma and Burn Surgery, Stroger Hospital of Cook County, Rush University, 8th floor, 1950 West Polk Street, Chicago, IL 60612 USA

**Keywords:** WSES, Open pelvic fracture, Blunt abdomen trauma

## Abstract

**Background:**

Open pelvic fractures are rare but complex injuries. Concomitant external and internal hemorrhage and wound infection-related sepsis result in a high mortality rate and treatment challenges. Here, we validated the World Society Emergency Society (WSES) classification system for pelvic injuries in open pelvic fractures, which are quite different from closed fractures, using the National Trauma Data Bank (NTDB).

**Methods:**

Open pelvic fracture patients in the NTDB 2015 dataset were retrospectively queried. The mortality rates associated with WSES minor, moderate and severe injuries were compared. A multivariate logistic regression model (MLR) was used to evaluate independent factors of mortality. Patients with and without sepsis were compared. The performance of the WSES classification in the prediction of mortality was evaluated by determining the discrimination and calibration.

**Results:**

A total of 830 open pelvic fracture patients were studied. The mortality rates of the mild, moderate and severe WSES classes were 3.5%, 11.2% and 23.8%, respectively (*p* < 0.001). The MLR analysis showed that the presence of sepsis was an independent factor of mortality (odds of mortality 9.740, *p* < 0.001). Compared with patients without sepsis, those with sepsis had significantly higher mortality rates in all WSES classes (minor: 40.0% vs. 3.1%, *p* < 0.001; moderate: 50.0% vs. 9.1%, *p* < 0.001; severe: 66.7% vs. 22.2%, *p* < 0.001). The receiver operating characteristic (ROC) curve showed an acceptable discrimination of the WSES classification alone for evaluating the mortality of open pelvic fracture patients [area under curve (AUC) = 0.717]. Improved discrimination with an increased AUC was observed using the WSES classification plus sepsis (AUC = 0.767).

**Conclusions:**

The WSES guidelines can be applied to evaluate patients with open pelvic fracture with accurate evaluation of outcomes. The presence of sepsis is recommended as a supplement to the WSES classification for open pelvic fractures.

## Introduction

Pelvic fracture is indicative of high-energy trauma with high mortality rates, which range from 5 to 16% [1]. The causes of death of pelvic fracture include uncontrolled retroperitoneal hemorrhage, associated intra-abdominal injuries or associated injuries of other body regions [2–4]. Compared with closed pelvic fractures, open pelvic fractures are relatively rare but more complex because of concomitant internal and external hemorrhaging [5]. In addition, open pelvic fracture is usually associated with soft tissue disruption and direct communication between the fracture site and the environment [6, 7]. Uncontrolled infection may result in sepsis and contribute to mortality [8–10]. Sepsis is associated with only 2–4% of all pelvic fractures but with a mortality rate of up to 45% [11–13]. Therefore, open pelvic fracture can be thought of as a specific pattern of pelvic fractures with quite different injury severities and strategies for treatment.

The conventional classification systems for pelvic fractures, including the Tile classification system and Young–Burgess classification system, evaluate pelvic fractures based on mechanical fracture patterns [14, 15]. However, in addition to mechanical instability, high-energy impact-related hemorrhage may result in unstable hemodynamics in pelvic fracture patients [16–18]. Hence, the World Society of Emergency Surgery (WSES) comprising surgeons and experts around the world published a novel classification system for pelvic injury in 2017 [19]. Both mechanical stability and hemodynamic stability were taken into consideration in these guidelines. This system provides a precise evaluation of patients with pelvic injuries.

In the current study, an international validation of the WSES classification for open pelvic fractures was performed. The National Trauma Data Bank (NTDB), which serves as the largest data bank of traumatic injuries and outcomes in the USA, was used as the studied sample to evaluate whether this system is applicable and precise for such a specific group of pelvic injuries. Furthermore, since sepsis is a specific complication among open pelvic injury patients due to open wound contamination, soft tissue loss and probably associated rectal injuries, the role of sepsis in the WSES guidelines was also discussed.

## Methods

### A priori hypothesis

We hypothesized that the WSES classification system could be applied to patients with open pelvic fractures, which are quite different from closed pelvic fractures. This classification system was presumed to accurately predict the outcomes of open pelvic fractures.

### Dataset and time window

We retrospectively queried the research datasets of the NTDB 2015.

### Inclusion/exclusion criteria

**Fig. 1:**
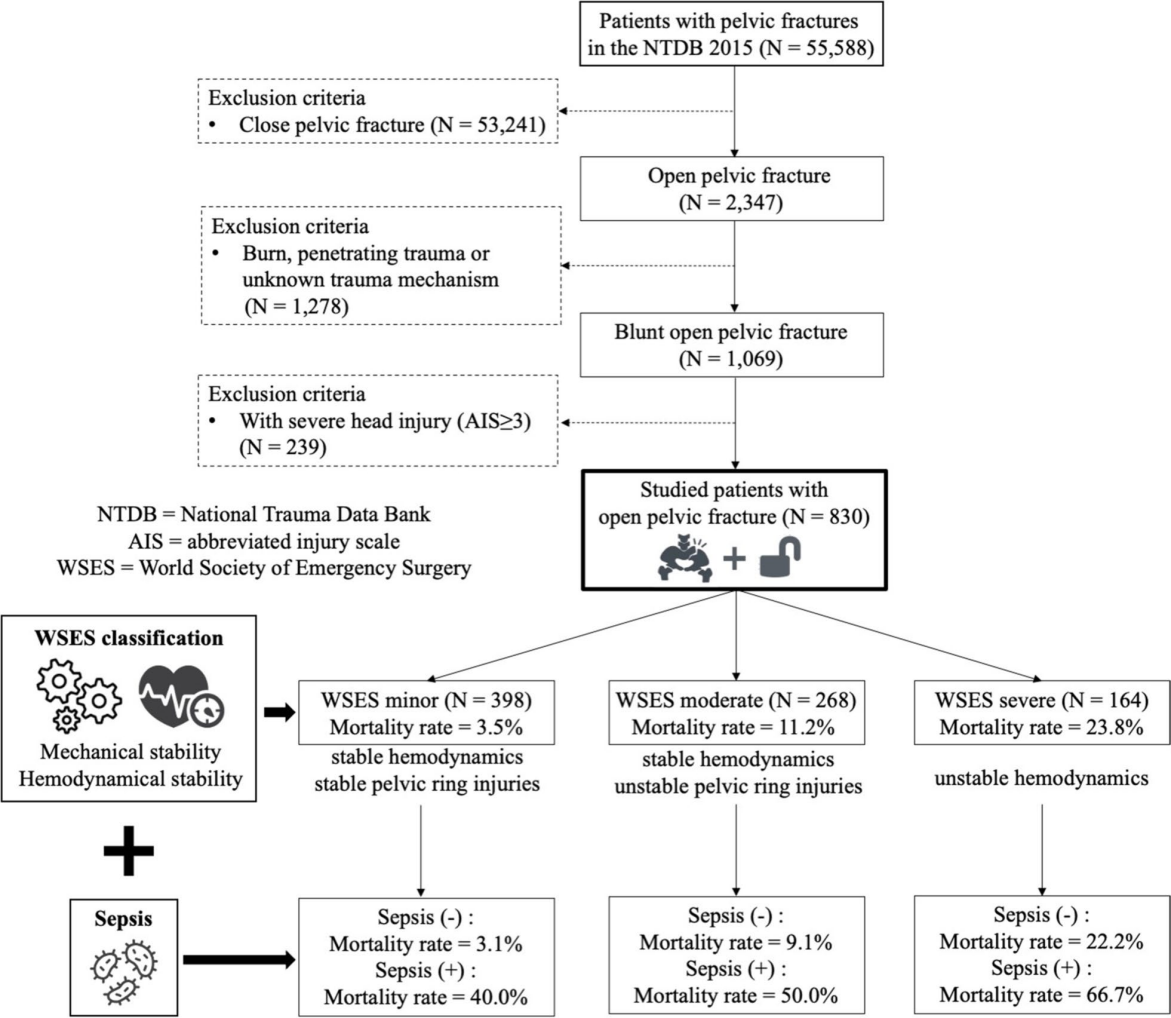
The enrollment of studied patients and study protocol of the current study

Patients with blunt and open pelvic fractures [International Classification of Diseases, Ninth Edition, Clinical Modification, (ICD)-9-CM codes: 808.1, 808.3, 808.5, 808.5x, 808.9] were included in the current study. The exclusion criteria were patients with non-blunt trauma (penetrating trauma, burns, other or unknown trauma mechanisms) or patients with severe head injuries [abbreviated injury scale (AIS) of the head score ≥ 3] that may increase the non-pelvic injury-related mortality [20]. The enrollment of the studied patients and the study protocol are shown in Fig. 1.

### Study setting

Open pelvic fracture patients were classified per the WSES guidelines [minor pelvic injuries: patients with stable hemodynamics and stable pelvic ring injuries; moderate pelvic injuries: patients with stable hemodynamics and unstable pelvic ring injuries (ICD-9-CM: 808.43, 808.53); severe pelvic injuries: any patients with unstable hemodynamics]. Unstable hemodynamics was defined as systolic blood pressure < 90 mmHg, pulse > 120 or use of a vasopressor. Other covariables in the current study included (1) unstable and stable pelvic ring injuries, (2) the presence and absence of associated vascular injuries (ICD-9-CM: 902.xx), and (3) the presence and absence of sepsis, which was defined in the glossary of the NTDB [severe sepsis (key = 32)].

### Outcome measurements

The mortality of patients with open pelvic fractures was the primary outcome of the current study. The secondary outcomes included hospital length of stay (LOS) and intensive care unit (ICU) LOS.

### Study design

The mortality rates of open pelvic fracture patients who were classified as having minor, moderate and severe injuries per the WSES classification were compared. When the mortality of patients with minor injuries was defined as a baseline, the odds of mortality in moderate injury patients and severe injury patients were calculated using logistic regression analysis. After excluding nonsurvivors, the hospital LOS and ICU LOS were compared among patients with different injury severities (minor, moderate and severe injuries).

For all open pelvic fracture patients, the WSES classification, presence of sepsis or not, presence of unstable pelvic ring injury and presence of associated vascular injury of survivors and nonsurvivors were compared. A subsequent multivariate logistic regression (MLR) model was performed using the “enter method” to evaluate independent factors of mortality. Statistically significant variables in the univariate analysis were included in the model except the presence of unstable pelvic ring injury because of multicollinearity (variance inflation factor value is > 10) between the WSES classification and the presence of unstable pelvic ring injury. (The unstable pelvic ring injury was originally included in the evaluation of the WSES classification.)

The characteristics and outcomes of all patients with and without sepsis were compared. Furthermore, the mortality rates were compared between these two groups when open pelvic fractures were stratified by the WSES classification. Finally, the performance of the WSES classification and the WSES classification plus the evaluation of sepsis in the evaluation of the mortality of open pelvic fracture patients was assessed by determining the discrimination and calibration. Discrimination was measured by calculating the area under the receiver operating characteristic (ROC) curve (AUC) [21, 22]. Calibration was assessed using the Hosmer–Lemeshow Ĉ test (with *p* > 0.05 indicating no significant difference between the predicted and observed outcomes) [21].

### Statistical analysis

R software version 3.5.0 from R Core Team (R Foundation for Statistical Computing, Vienna, Austria, 2018) and R Studio software version 1.1.453 from R Studio: Integrated Development for R (R Studio, Inc., Boston, Massachusetts, 2016) were used to analyze the original files from the NTDB [23]. Chi-square tests were used to compare the nominal data, which are presented as numbers and percentages. Student’s t tests or analysis of variance (ANOVA) tests were used to compare the numerical data, which are presented as the means with standard deviations. Statistical significance was defined as a value of *p* < 0.05.

## Results

During the one-year study period, there were 55,588 pelvic fracture patients in the NTDB [2,347 (4.2%) of them had open pelvic fractures and 53,241 (95.8%) of them had closed pelvic fractures]. After excluding patients who met the exclusion criteria (non-blunt trauma or severe head injury), a total of 830 open pelvic fracture patients were studied. Among these patients, the numbers and proportions of WSES minor injury, WSES moderate injury and WSES severe injury were 398 (48.0%), 268 (32.3%) and 164 (19.7%), respectively. Among all studied patients, 25 (3.0%) patients had sepsis. The overall mortality rate was 10.0% (*N* = 83) (Fig. 1).

The mortality rates and associated odds of mortality of open pelvic fracture patients with different WSES severities are shown in Table 1. These values increased significantly as the injury severity increased from minor injury to severe injury (mortality rate: 3.5% for minor injuries, 11.2% for moderate injuries and 23.8% for severe injuries, *p* < 0.001; odds of mortality 3.457 for moderate injuries and 8.558 for severe injuries with minor injuries as the baseline, *p* < 0.001). After excluding nonsurvivors, the hospital LOS and ICU LOS also increased significantly from minor injury to severe injury (hospital LOS: 12.0 days for minor injuries, 17.8 days for moderate injuries and 18.5 days for severe injuries, *p* < 0.001; ICU LOS: 3.2 days for minor injuries, 6.8 days for moderate injuries and 6.8 days for severe injuries, *p* < 0.001).

**Table 1:** Outcomes (mortality rate, hospital LOS and ICU LOS) among different WSES classes of open pelvic fractures in the NTDB 2015 (*N* = 830)

Outcome	Minor (*N* = 398)	Moderate (*N* = 268)	Severe (*N* = 164)	*p* value
Mortality				
Mortality rate (*N*, %)	14 (3.5%)	30 (11.2%)	39 (23.8%)	< 0.001*
Odds of mortality	– (baseline)	3.457 (1.797–6.654)	8.558 (4.498–16.281)	< 0.001^†^
LOS (days) (excluding mortality patients)				
Hospital LOS	12.0 (10.6–13.5)	17.8 (14.6–21.0)	18.5 (14.9–22.1)	< 0.001^‡^
ICU LOS	3.2 (2.5–4.0)	6.8 (5.3–8.4)	6.8 (5.0–8.6)	

Mortality rate: *N* (percentage), odds of mortality: odds (95% confidence interval)

Hospital LOS: mean (95% confidence interval), ICU LOS: mean (95% confidence interval)

LOS, length of stay; ICU, intensive care unit; WSES, World Society of Emergency Surgery; NTDB, National Trauma Data Bank

^*^Chi-square test; ^†^Logistic regression; ^‡^ANOVA (analysis of variance)

Compared with survivors, nonsurvivors had significantly higher proportions of moderate injuries (36.1% vs. 36.9%, *p* < 0.001), severe injuries (47.0% vs. 16.7%, *p* < 0.001), presence of sepsis (15.7% vs. 1.6%, *p* < 0.001), presence of unstable pelvic ring injury (65.1% vs. 41.2%, *p* < 0.001) and presence of associated vascular injury (22.9% vs. 9.8%, *p* < 0.001) (Table 2). The subsequent MLR analysis showed that the presence of sepsis was an independent risk factor for the mortality of open pelvic fracture patients after adjusting for the WSES classification (odds of mortality: presence of sepsis 9.740, *p* < 0.001) (Table 3).

**Table 2:** Comparisons between nonsurvivors and survivors with open pelvic fractures (*N* = 830)

Variable	Nonsurvivors (*N* = 83)	Survivors (*N* = 747)	*p* value
WSES classification			< 0.001^†^
Minor (*N* = 398)	14 (16.9%)	384 (51.4%)	
Moderate (*N* = 268)	30 (36.1%)	238 (31.9%)	
Severe (*N* = 164)	39 (47.0%)	125 (16.7%)	
Sepsis (*N*, %)	13 (15.7%)	12 (1.6%)	< 0.001^†^
Unstable pelvic ring injury (*N*, %)	54 (65.1%)	308 (41.2%)	< 0.001^†^
Associated vascular injury (*N*, %)	19 (22.9%)	73 (9.8%)	< 0.001^†^

Nominal data: *N* (percentage)

WSES, World Society of Emergency Surgery

^†^Chi-square test

**Table 3:** Multivariate logistic regression analysis of the evaluation of independent risk factors for mortality in open pelvic fracture patients (*N* = 830)

Variable	*p* value*	Odds of mortality	95% confidence interval	
			Lower	Upper
WSES classification				
Minor	–	– (baseline)	–	–
Moderate	0.002	2.892	1.474	5.675
Severe	< 0.001	7.875	4.062	15.268
Sepsis	< 0.001	9.740	4.001	23.709
Associated vascular injury	0.163	–	–	–

^*^Multivariate logistic regression

WSES = World Society of Emergency Surgery

**Fig. 2:**
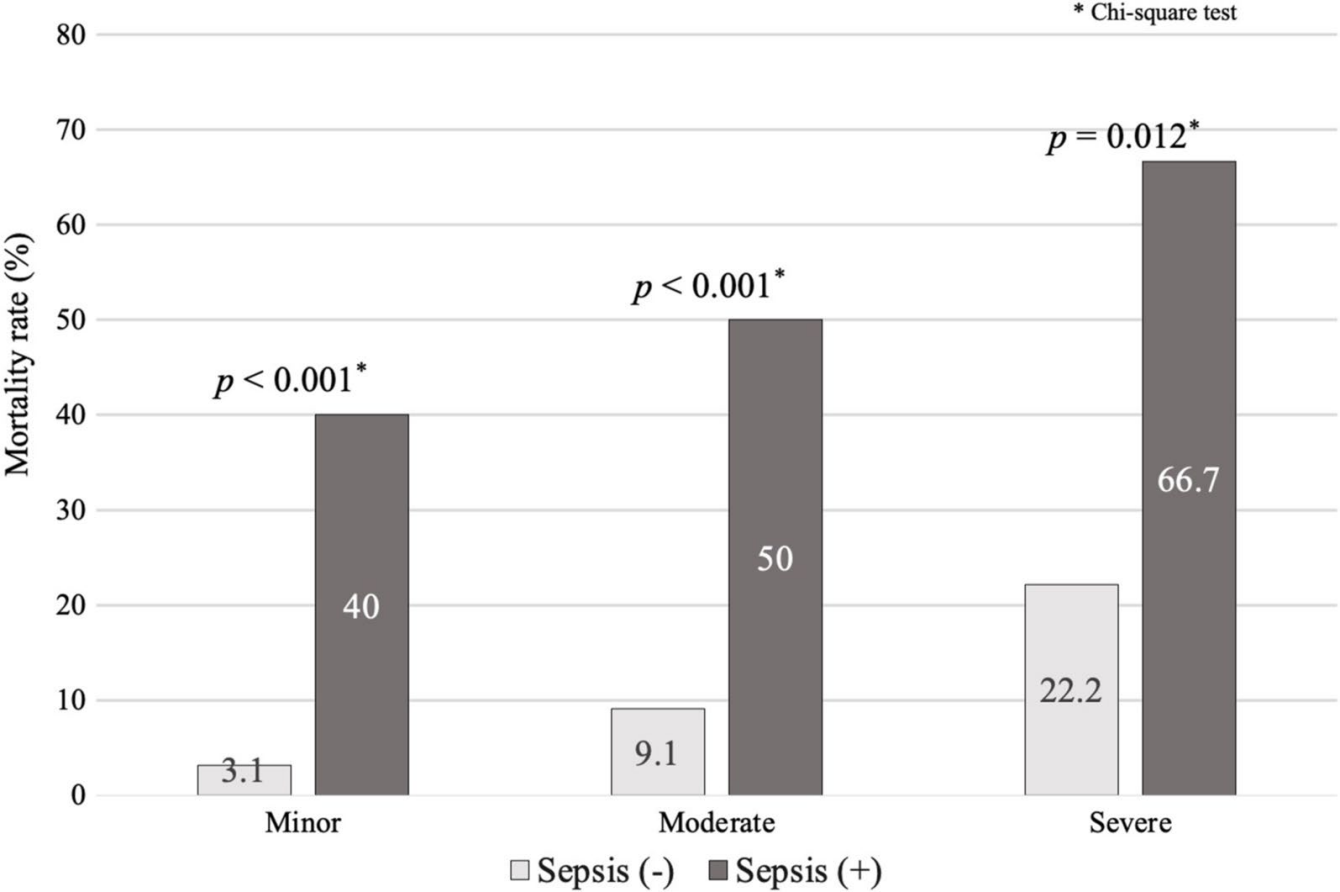
Comparisons of mortality rates between open pelvic fracture patients with and without sepsis in WSES minor, moderate and severe injuries

The comparisons between open pelvic fracture patients with and without sepsis are shown in Table 4. Patients with sepsis had a significantly higher mortality rate (52.0% vs. 8.7%, *p* < 0.001), a longer hospital LOS (34.6 vs. 13.4 days, *p* < 0.001) and a longer ICU LOS (21.1 vs. 4.5 days, *p* < 0.001) than patients without sepsis. In addition, patients with sepsis had significantly higher proportions of unstable pelvic rings (72.0% vs. 42.7%, *p* < 0.001) and associated vascular injury (32.0% vs. 7.6%, *p* < 0.001) than patients without sepsis. After stratification based on the WSES classification, sepsis patients had significantly higher mortality rates than nonsepsis patients in each class (minor injuries: 40.0% vs. 3.1%, *p* < 0.001; moderate injuries: 50.0% vs. 9.1%, *p* < 0.001; severe injuries: 66.7% vs. 22.2%, *p* < 0.001) (Fig. 2). Among all nonsurvivors (*N* = 83), 60.2% (*N* = 50) died within 2 days. However, patients with sepsis (*N* = 13) had a significantly longer time to mortality than patients without sepsis (*N* = 70) (17.2 vs. 4.2 days, *p* < 0.001).

**Table 4:** Comparisons between open pelvic fracture patients with and without sepsis (*N* = 830)

Variable	Sepsis (+) (*N* = 25)	Sepsis (−) (*N* = 805)	*p* value
Unstable pelvic ring injury (*N*, %)	18 (72.0%)	344 (42.7%)	0.004^†^
Associated vascular injury (*N*, %)	8 (32.0%)	61 (7.6%)	< 0.001^†^
Outcomes			
Mortality (*N*, %)	13 (52.0%)	70 (8.7%)	< 0.001^†^
Hospital LOS (days)	34.6 (22.0–47.2)	13.4 (12.2–14.7)	< 0.001*
ICU LOS (days)	21.1 (16.4–25.8)	4.5 (3.9–5.1)	< 0.001*

Numerical data: mean (95% confidence interval); nominal data: *N* (percentage)

^†^Chi-square test; *Student’s t test

**Fig. 3:**
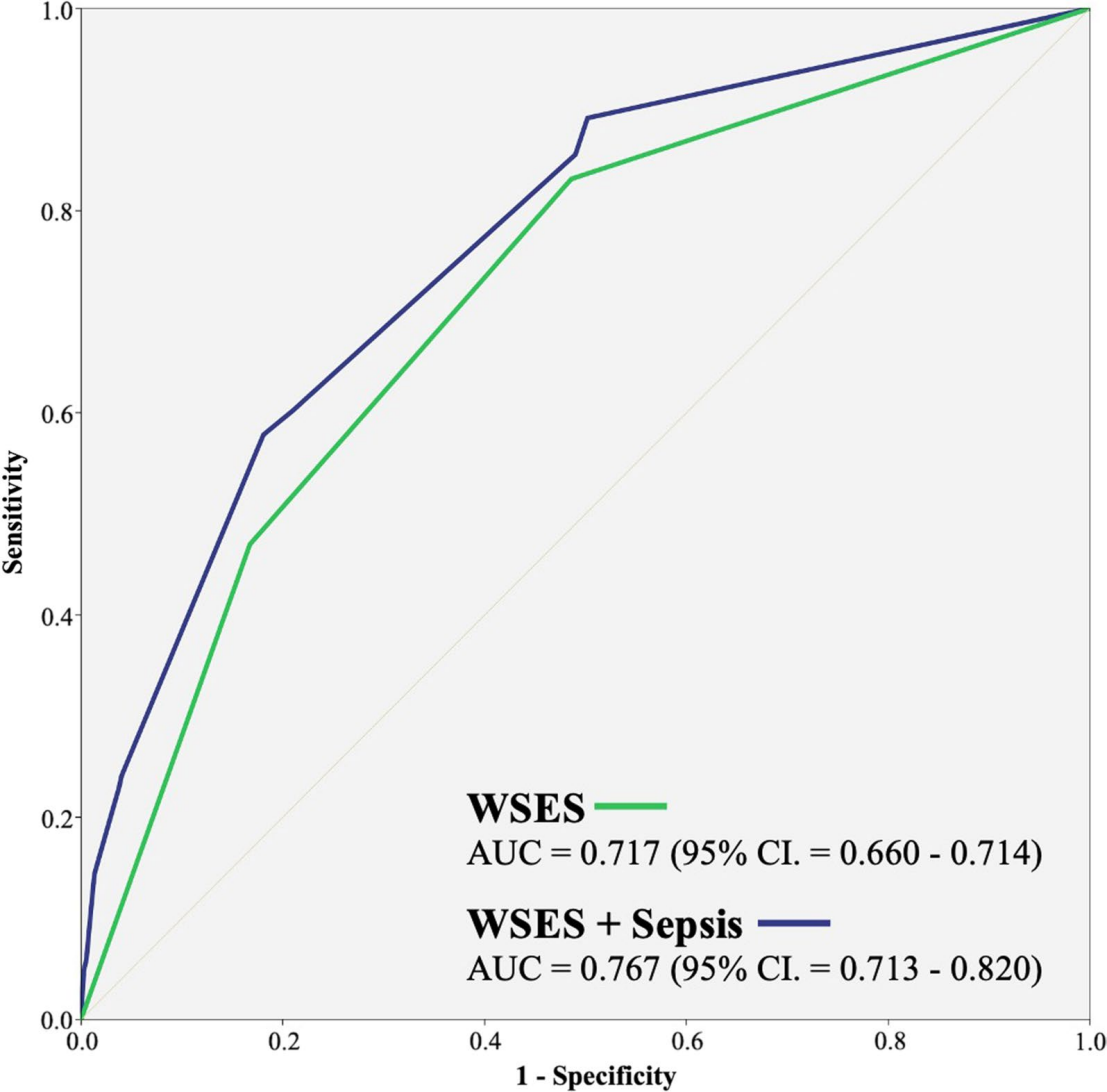
Discrimination performance of the WSES classification and the WSES classification plus sepsis for the prediction of mortality in open pelvic fracture patients [data are presented with the receiver operating characteristic curve, along with the area under the curve (AUC) and 95% confidence interval (CI)]

The WSES classification system showed acceptable discrimination in predicting mortality associated with open pelvic fractures, with an AUC of 0.717 (95% confidence interval 0.660–0.714) and good calibration, with the Hosmer–Lemeshow Ĉ test showing *p* = 1.000. After adding sepsis as another covariable (WSES guidelines + sepsis), a better performance in predicting mortality associated with open pelvic fractures was observed [AUC = 0.767 (95% confidence interval 0.713–0.860) and Hosmer–Lemeshow Ĉ test *p* = 0.828] (Fig. 3).

## Discussion

### International validation of the WSES classification for open pelvic fracture patients

Bony structure instability and internal hemorrhage can mostly be conservatively managed in closed pelvic fracture patients; however, the management of patients with open pelvic fractures can be challenging and requires a multidisciplinary approach. In addition to stopping concomitant external and internal hemorrhage, the difficulty of open pelvic fracture treatment includes the management of severe wound infection and further sepsis and multiple organ failure [11, 24]. The WSES guidelines suggest that the management of pelvic trauma must consider physiological and mechanical derangement [17, 19]. An international validation using the National Trauma Data Bank proved that the WSES guidelines were an accurate and reproducible classification system for pelvic injuries [18]. However, for open pelvic fractures, which are more dangerous than closed fractures, it is important to evaluate whether the WSES guidelines are still accurate and applicable.

In the current study, the accuracy of the WSES classification system for evaluating mortality in open pelvic fracture patients was validated by using the NTDB. The mortality rates associated with minor injury, moderate injury and severe injury were 3.5%, 11.2% and 23.8%, respectively. The odds of mortality also increased significantly from minor injury to severe injury (Fig. 1 and Table 1). Moreover, after excluding mortality patients, the hospital LOS also increased significantly as the class of injury increased from minor injury to severe injury in open pelvic injury patients. (Most patients with severe injuries may die within a few days because of uncontrolled hemorrhage or other associated injuries. Therefore, it is unfair to evaluate the LOS using all studied patients with both survivors and nonsurvivors.) Although there are differences between open and closed pelvic fractures, this nationwide study showed that the WSES guidelines still serve as a good evaluation modality.

### The role of sepsis in open pelvic fractures and the WSES classification system

In addition to controlling hemorrhage, another challenge in the management of open pelvic fractures is the treatment of wound infection and further sepsis [25, 26]. A previous study reported that 21.4% of open pelvic fracture patients had colorectal injuries and 14.3% of patients had genitourinary system injuries [5]. Associated anorectal or urogenital injuries, contaminated soft tissue injuries and wound-related infection may lead to several complications, such as septicemia, coagulopathy, multiple organ dysfunction, hypotension and mortality [24]. The management of open pelvic fracture infection includes broad-spectrum antibiotics for both gram-positive and gram-negative bacteria, surgical debridement with presacral and perianal drainage and early colostomy for stool diversion [9, 27–29].

Therefore, we recognize that the evaluation of outcomes of open pelvic fracture cannot be limited to the status of hemorrhage or hemostasis, but infection and sepsis should also be considered. In 1997, the Jones–Powell classification system was developed to evaluate the morbidity and mortality of patients with open pelvic fracture [28]. In addition to mechanical stability, the concept of rectal injury evaluation and early diverting colostomy for infection control was considered in the classification system. However, this classification system was developed based on only a small number of patients (*N* = 39). A subsequent multicenter study that validated the Jones–Powell classification system in 2013 only had 64 patients [30]. Herein, many patients with open pelvic fractures in a nationwide databank were studied. Among all classes of injury severity, the mortality rates of patients with sepsis were significantly higher than those of patients without sepsis (Fig. 2). Furthermore, the proportion of sepsis in nonsurvivors was significantly higher than that in survivors (15.7% vs. 1.6%, *p* < 0.001). The subsequent MLR showed that sepsis served as an independent factor for the mortality of open pelvic fracture patients after adjusting for the WSES classification (Table 3).

The ROC curve showed an acceptable discrimination of the WSES classification alone for evaluating the mortality of open pelvic fracture patients (AUC = 0.717). However, an improved discrimination with an increased AUC was observed using the WSES classification plus sepsis (AUC = 0.767). In other words, sepsis plays an important role in the mortality of open pelvic fractures. Hence, the evaluation of sepsis is recommended as a supplement to the WSES classification for open pelvic fracture, which is associated with soft tissue injury and wound infection.

### The association between hemorrhage and sepsis in patients with open pelvic fractures

It has been suggested that the cause of death among open pelvic fracture patients could be classified as death related to hemorrhage or associated injuries and death related to sepsis and further multiple organ failure [9, 11]. Uncontrolled hemorrhage-related mortality usually occurs within days of arrival at the emergency department because of the failure of hemostatic procedures (angioembolization or surgery). On the other hand, after the achievement of hemostasis, sepsis may occur and result in delayed mortality [1, 7]. In the current study, although most mortality (*N* = 50, 60.2%) occurred within 2 days, the mean LOS of mortality patients with sepsis was significantly longer than patients without sepsis (17.2 vs. 4.2 days, *p* < 0.001). The results of the current study supported that sepsis was related to late mortality. During the early stage of open pelvic fracture management, it is vital to achieve effective hemostasis, whereas sepsis control and the treatment of multiple organ failure may require efforts to prevent late mortality.

In patients with open pelvic fracture, the soft tissue injury from crush impact and hemorrhage from a hemostatic procedure may impair tissue perfusion, and the open contaminated wound may aggravate the infection condition. Previous reports that discussed the WSES guidelines for pelvic fractures suggested the evaluation of associated vascular injuries [17, 18]. The role of vascular injuries is more significant than the mechanical stability of the pelvis in the mortality associated with pelvic fractures. The current study found that the associated vascular injury increased the probability of sepsis in patients with open pelvic fractures. Therefore, concomitant vascular injury and sepsis may synergistically negatively affect each other in patients with open pelvic fractures.

### Limitations of the current study

Previous studies of open pelvic fractures usually had small patient numbers [5, 7, 25, 28]. The advantage of the NTDB is its large number of relatively rare injuries; the significance of analyses could be augmented accordingly. However, the retrospective nature and possibly inaccurate records of the NTDB could limit the power of evidence. Furthermore, the definition of unstable hemodynamics in the current study was based on the vital signs upon emergency department arrival. The response to resuscitation could not be evaluated. Moreover, patients with open pelvic fractures are usually polytraumatized [2–4]. Patients with isolated open pelvic fractures were very rare. The associated injuries may affect the evaluation of mortality using the WSES classification system. Finally, the NTDB 2015, which is not an updated dataset, was used in the current study. However, the details of treatment that may be advanced in recent years were not discussed. We believe that the data from 2015 are applicable for an observational study only.

## Conclusion

The WSES guidelines can be applied to evaluate patients with open pelvic fractures with accurate evaluation of outcomes. The presence of sepsis is recommended as a supplement to the WSES classification for open pelvic fractures.

## Data Availability

National Trauma Data Bank.

## Abbreviations

WSES: World Society Emergency Society
NTDB: National Trauma Data Bank
MLR: Multivariate logistic regression
ROC: Receiver operating characteristic
AUC: Area under curve
CI: Confidence interval
ICD-9-CM: International Classification of Diseases, Ninth Edition, Clinical Modification
AIS: Abbreviated injury scale
LOS: Length of stay
ICU: Intensive care unit
ANOVA: Analysis of variance
